# The learning of sprint hurdles: A comparative study on increasing contextual interference and blocked practice schedules

**DOI:** 10.1371/journal.pone.0289916

**Published:** 2024-01-10

**Authors:** Hubert Makaruk, Beata Makaruk, Marcin Starzak, Kazimierz Chmielewski, Jared M. Porter

**Affiliations:** 1 Department of Athletics, Faculty of Physical Education and Health in Biala Podlaska, The Józef Piłsudski University of Physical Education in Warsaw, Biała Podlaska, Poland; 2 Department of Physical Education Methodology, Faculty of Physical Education and Health in Biala Podlaska, The Józef Piłsudski University of Physical Education in Warsaw, Biała Podlaska, Poland; 3 Department of Kinesiology, Recreation, and Sport Studies, The University of Tennessee, Knoxville, TX, United States of America; University of Illinois at Urbana-Champaign, UNITED STATES

## Abstract

The contextual interference (CI) approach has proposed that a random order of practice for motor skills is superior in facilitating learning compared to a blocked arrangement of practice trials. Two groups of physical education students learned sprint hurdles, employing either an increasing CI practice schedule (n = 23) or a blocked practice schedule (n = 23). In both the practice schedules, the same exercises were used in a different trial order during each learning session. Eleven practice sessions were conducted over a period of six weeks, with two days of practice per week. Ten and 40 days after the acquisition phase, a retention and transfer test were conducted. The results showed no differences between the two practice schedules during the retention tests. However, students practicing with an increasing CI arrangement performed better on the delayed transfer test compared to students which practiced with a blocked schedule. Specifically, the increasing CI group more effectively (*p* < 0.05) cleared the hurdles due to a lower take-off step angle and longer step length than the blocked practice group. Although utilizing an increase in CI during the learning phase of sprint hurdling produced more persistent learning effects relative to a traditional blocked practice schedule for adult novice learners, further research is warranted to explore the CI effect across a broader range of sport skills.

## Introduction

One primary goal of physical education (PE) teachers is to teach motor skills [1], which are defined as activities requiring voluntary control over movements of the joints and body segments to achieve a goal. This capability for physical educators is clearly emphasized by many national standards, for example “A physically educated person demonstrates competency in motor skills and movement patterns needed to perform a variety of physical activities” [2]. Previous research shows that motor skill acquisition is greatly improved when a demonstration of the to be learned skill occurs before practicing the task compared to only providing verbal instructions prior to the skill being physically practiced [3]. Interestingly, a study by Baghurst et al. [4] reported that many PE teacher education programs do not require demonstrating proficiency in sport skill technique before graduation. The lack of effectively evaluating motor skill execution prior to graduating from a PE teacher preparation program may negatively influence motor skill learning by students taught by teachers which have not acquired the capabilities to properly execute skills they are expected to teach at a later time. Therefore, there is a need to find optimal solutions for teaching and learning sport skills in programs for PE students aspiring to be future PE teachers. From the perspective of a program’s learning outcomes, it is highly desirable that mastery of learned skills be relatively permanent, stable, and learners possess the ability to adapt a skill to novel variations or contexts [5].

There are many established methods which can be utilized to effectively learn motor skills. One of the most widely examined strategies is the adoption of practicing with higher rather than lower amounts of contextual interference (CI). Research has shown that learning outcomes may be greater and longer-lasting when related motor skills are practiced in a randomized order (high CI) compared to practicing within repetitive same skill blocks (low CI) of trials [6]. The learning benefit which is the product of practicing tasks in a trial arraignment which produces greater rather than lower amounts of CI is known as the CI effect [7]. It is worth noting here that a negative influence of interference is often found for high CI schedules during the acquisition phase or the initial phase of practice. For example, Wrisberg and Liu [8] investigated the effects of blocked and random practice when multiple badminton serves were being learned. Students in the blocked practice group performed all their trials with one type of serve in a repeating pattern (low CI) before they practiced a new service variation. Whereas, the random group of students practiced all types of serves in an alternating fashion (high CI). There were no differences in acquisition, but the random group achieved significantly higher scores on the short serve in the retention test compared to the group of participants which practiced with low CI. In addition, short and long serves of the high CI group were significantly better in the transfer tests compared to the group of adult beginners which practiced with low CI. Findings of another study [9], where learning of snowboarding skills in PE students was investigated demonstrated that random practice resulted in enhanced performance during acquisition and retention compared to a group of PE students practicing the same skills in a blocked order of trials.

Although a number of studies have confirmed the existence of a CI effect on motor skill learning [10], other studies have not consistently replicated this finding. For example, random practice did not lead to greater learning effects in students who practiced basic volleyball skills compared to those using blocked practice [11]. Moreover, the typical CI effect did not occur when students practiced basketball [12] or soccer [13] related skills. Some researchers have reported that high CI may overwhelm novice learners. Specifically, if the amount of CI is too high during practice, then the benefits of this form of practice may not immerge on delayed retention and transfer tests [14]. To address this concern in novice learners Porter and Magill [15] proposed an alternative form of practice which introduced the novice to systematic increases in CI. Their study conducted experiments to compare blocked and random practice schedules with a form of practice that exposed the learner to increasing CI throughout the acquisition phase of practice [15]. The increasing practice schedule began with blocked (low CI) scheduling, followed by a serial (moderate CI) order of practice trials. Practice then concluded with a random (high CI) arrangement of practice trials. The results revealed that novice students who utilized increasing CI during acquisition learned the task better than equally skilled learners that practiced with fixed blocked or random schedules. Learning advantages for the increasing CI over blocked and random practice have also been shown for basketball passing tasks in moderately skilled male college-aged students that practiced over multiple days [16].

The concept of increasing CI is consistent with multiple motor learning principles, including the learning stages model offered by Gentile [17] and the Challenge Point hypothesis by Guadagnoli and Lee [18]. Porter and Magill [15] suggested the difficulty within a practice environment should be systematically increased because the level of skill within the learner increases during the skill acquisition process. Thus, by consistently challenging learners at an evolving relevant level, an optimal learning environment is created. The Challenge Point hypothesis proposes that difficulty within a practice environment is a function of the relationship between the task difficulty which is a constant (i.e., nominal task difficulty) regardless of the learner or practice environment, and functional task difficulty which is relative to the skill level of the learner. Another explanation for the observed benefits of increasing CI is consistent with Bjork’s [19, 20] idea of “desirable difficulties” which suggests that optimal learning is the product of exposing the learner to appropriate difficulties during practice. That is, if a learner experiences a level of difficulty that is too high or too low during practice, learning will be compromised. The work first demonstrated by Porter and Magill [15] suggest that practicing with increases in CI is an effective way to create an appropriate challenge point and a desirable difficulty to facilitate motor learning.

The potential for enhancing the learning of sport skills by increasing CI during practice has also been demonstrated in the development of volleyball skills in university aged students [21] as well as in badminton [22] with children. However, with such a limited number of studies published on the topic and some conflicting results [23] it is premature to conclude the long-term effects this schedule may offer for students in a PE setting. Therefore, further research in this field is needed. It would be best for ecological validation of the learning method if new solutions utilizing CI were tested under applied conditions within university classes which have a limited number of class periods for practice, and limited availability for teacher feedback because of a high teacher to student ratio. To achieve this goal we investigated the learning of sprint hurdles under real-world conditions within a PE course. Learning sprint hurdles is a common component within a track and field PE curriculum. The purpose of this skill is to cover a specified distance while clearing the hurdles as fast as possible. The traditional approach to practicing sprint hurdles, as well as other track and field events, is typically through the adoption of a blocked practice schedule [24]. This is likely due to the fact that sprint hurdles is a closed skill for which the environmental conditions such as running distance, hurdle height, and distance between hurdles are fixed. Hence it may seem logical that repetition-oriented (i.e., blocked) training which offers a high degree of stability in consecutive trials would be most effective for learning. In addition, a blocked schedule is easier to incorporate within a practice setting because it does not require continuous modifications of each subsequent exercise, as is the case in a highly variable random schedule. It also seems that the immediacy of visible performance outcomes during acquisition may also persuade instructors to implement a blocked form of practice within their classes. Additionally, the high skill complexity of sprint hurdling is another barrier that may discourage teachers from utilizing high CI in learning this particular skill. Especially since some authors suggest if a skill is relatively complex for the performer, the amount of CI will likely have minimal impact on the amount of expended cognitive effort [10]. Finally, the more stable practice conditions offered by a blocked practice schedule for beginners may more effectively counteract the fear of overcoming a hurdle with high speed. Considering these constraints and the potential limitations of the CI effect, it appears that increasing CI during practice may be a particularly beneficial form of practice to learn sprint hurdling.

The present experiment was designed to determine if increasing CI during practice provided advantages relative to traditional (i.e., blocked practice) learning of sprint hurdles in university students who participated in a track and field PE course. The teaching of movement skills to students preparing to become PE teachers is carried out under specific conditions, often within narrow timeframes, numerous groups of students practicing together, and accompanying additional movement exercises such as a warmup and cool down. Therefore, the search for effective solutions, which are relatively simple to implement, and produce stable learning outcomes, which are validated in practical conditions, are highly desirable. Given the strong, but not conclusive theoretical underpinnings of the CI effect in learning sport skills [10], we expected to see advantages in the kinematics of hurdle jumping in near (10-day delayed) and far (40-day delayed) retention and transfer testing when practicing with an increasing amount of CI relative to equally skilled learners practicing the same task in a traditional blocked arrangement. Observing differences in jump kinematics is a primary dependent measure in the present study considering the participants are learning proper jumping technique so they can teach and demonstrate the skills to their future PE students once they become a PE teacher.

## Material and methods

### Material

Forty-six physically active male undergraduate students (mean age = 21.3 years, s = 0.8 years) volunteered to participate in this experiment. A power analysis (G*Power 3.1) was conducted to ensure the appropriateness of the sample size. The calculation with an estimated moderate effect size reported for a Group x Time interaction with the following statistical assumptions: α = 0.05, β = 0.95, effect size (ƒ = 0.25), number of groups = 2, number of measurements = 2, correlation among repetitions measures = 0.7, showed that a minimum of 34 participants would be required. Inclusion criteria for participation in the study were: a) healthy male PE students who completed 60 h of track and field as part of their university PE course; b) no previous experience with sprint hurdles; c) agreement to participate in the full study protocol; d) they were not injured or had surgery on a lower limb within six months prior to volunteering to participate in the present experiment. Participants were excluded if they did not attend both acquisition sessions and both testing sessions. All students were physically active for at least eight hours a week due to the nature of their participation in the PE teacher’s preparation curriculum. All were naïve to the research hypothesis being tested. Informed written consent was obtained from each volunteer before testing. The study was approved by a university Institutional Review Board.

### Task and apparatus

The task involved the 50 m sprint hurdles from a standard track starting block. All practice and testing sessions took place inside a climate controlled enclosed track and field hall. Participants were asked to initiate running for each trial following a hand clap which served as the “go” signal, and cover the distance of five hurdles with maximum speed. Before the trial, participants were informed they would receive the start commands “on your mark,” followed by “set” before hearing a start signal (a hand clap). The hurdles were placed on a synthetic track surface according to the requirements of the track and field course (see S1 File).

Since effective hurdle clearance is critical to achieve sprint hurdle mastery [25], the following hurdle step kinematics were analyzed in this study: ground contact time before hurdle clearance, hurdle flight time, hurdle step length, hurdle step velocity, and hurdle step take-off angle. Ground contact time before clearance of the hurdle was measured as the period from foot touchdown to the take-off of the same foot. Hurdle flight time was measured as the period from foot take-off to touchdown of the opposite foot while clearing the hurdle. Hurdle step length was determined as the distance from the tip of the shoe at take-off to the tip of the opposite leg’s shoe at take-off while clearing the hurdle. Hurdle step velocity was determined as the ratio between step length and the sum of the contact time of the pushing leg and flight time while clearing the hurdle. Step angle was defined as the angle of the parable tangent deriving from the movement of a step. The theoretical parabola for determining the step angle was calculated by using the step length and the maximum height of the foot during a step [26]. To evaluate sprint hurdle performance, the running time was also taken into consideration. Step angle was measured in degrees (deg), all other kinematics were measured in seconds (s).

Forty pairs of 1-m Optojump Next (Microgate, Bolzano, Italy) bars were placed parallel to each other along the track for the duration of the study. The measurement bars were positioned between 10 and 50 m of the covered distance. The Optojump Next bars transmit a solid infrared light beam to each other at a height of 2 mm above the running surface. Disruption of the infrared lights triggers a detector with a timing accuracy of 1 ms. In addition, the running time of each trial was recorded using portable timing lights (Witty timer, Microgate, Bolzano, Italy). Before commencing this study, the reliability of the hurdle step kinematics was evaluated using a test-retest methodology. The intraclass correlation coefficients (ICCs) of our reliability assessment were high (0.89–0.96).

In addition to collecting a variety of kinematic measures, we also evaluated sprint hurdling technique by using a rating scale which was designed and used by track and field university teachers to evaluate the learning outcomes of hurdling in a university course. One experienced teacher and a former track and field coach rated the hurdling performance of all trials during the retention and transfer tests. The outcome assessor was blinded to the group assignments. A four-point scale for evaluation of hurdling technique was adopted, where 3 represented criterion that was perfectly met, 2 was awarded when criterion was sufficiently met, 1 was scored when criterion was partly met and 0 when criterion was not met. The following items were assessed: (i) sprint start, (ii) the approach to the first hurdle, (iii) position and trajectory of the body during the hurdle clearance, including the action of the lead and trail legs and arms, and (iv) step pattern between hurdles. As a result, the mean scoring for all items was used for analysis. Prior to the study, two track and field teachers rated 18 independent trials of volunteers who were considered moderately skilled in sprint hurdles. Inter-rater reliability was independently assessed for each technique item by using the intraclass correlation coefficient (ICC). The ICC value for scored items ranged from 0.81 to 0.90, which is considered a high level of agreement.

### Procedure

This experimental program was conducted as part of a regular track and field course for university students studying to become a PE teacher. Each practice session started with a 20-min dynamic warm-up. This was followed by half of the students practicing hurdling (30 min), whereas the other half practiced the high jump (30 min). Following the initial 30 minute period, the practiced tasks were alternated so both groups had equal practice time per task. The experiment was divided into two stages. During the first stage, participants completed six practice sessions across three weeks (Monday and Friday) to become familiar with the sprint hurdling task. Each session involved the completion of the following basic hurdle exercises: hip mobility and flexibility exercises, wall exercises with the hurdle, walking over the hurdles, skipping beside the hurdles as a drill for the lead and trial legs, sprints over lying sticks and mini-hurdles (height: 0.3–0.6 m) with a decreasing number of steps from five to three steps between hurdles (see S2 File). The difficulty of the exercises was progressively increased by increasing the height and distance between obstacles. Each hurdle exercise followed a blocked order of trials for all participants. Specifically, a given exercise was repeated three times before the participants moved on to another exercise. Before beginning each exercise, the teacher described and demonstrated how to correctly perform each task. Major movement and technique errors were corrected immediately after each exercise by providing knowledge of performance feedback, e.g. (1) “clear the hurdle as quickly as possible”; (2) “clear the hurdle as flat as possible”; (3) “drive the lead leg immediately behind the hurdles”; (4) “drive the trail leg and hips further behind the hurdle”; (5) “run faster to the hurdle”; (6) “increase your step length when you run.” One feedback statement was given after each sprint attempt.

Before the second stage of the experiment, which occurred during the 7^th^ practice session, participants performed 50 m sprint hurdles as a pre-test. Following the pre-test, participants were stratified by the running time of the pre-test and then each participant was randomly placed into the Blocked (n = 23) or Increasing CI group (n = 23) with the constraint that there were equal numbers of participants in each group. Then, both groups completed five practice sessions utilizing the identical number of sprint hurdles and total distance, but with different sprinting distances (see S3 File). Students assigned to the Blocked group repeated sprint hurdles at the same distance with the same number of hurdles during each session. The distance of sprint hurdles and the number of the hurdles was increased every session, until the last practice session. In turn, the distances and number of hurdles during the practice schedule of the Increasing CI group were different in each of the subsequent practice sessions. Subjects rested from 3 to 6 minutes between sprint repetitions. As the running distance increased, so did the following rest period. Following the practice phase of the experiment, near (i.e., 10 day) and far (40 day) retention and transfer tests were conducted to assess motor learning of the sprint hurdling task.

### Statistical analyses

Descriptive statistics are presented as means ± SD. Data were tested for normality and homogeneity of variance assumptions. A 2 (Group) × 5 (Time) analysis of variance (ANOVA) with repeated measures on the second factor was used to assess potential differences between the Blocked and Increasing CI groups across the five sessions of practice (Time). Subsequently, the effect of the intervention on sprint hurdle performance was evaluated by using separate two-way ANOVAs with repeated measures on the second factor (Time) for the near (10 day) and far (40 day) retention and transfer tests. When significant effects were observed, a Tukey post hoc test was conducted. The level of statistical significance was set at *p* < .05. Effect sizes were calculated by partial eta squared (ƞ_p_^2^) and Cohen’s d for pairwise comparisons (d). Partial eta squared values were considered to be small (ƞ_p_^2^ = .01), moderate (ƞ_p_^2^ = .06) or large effects (ƞ_p_^2^ = .14) [27]. Cohen’s d was calculated with the following criteria: trivial (d = from 0 to .19), small (d = from .20 to .49), medium (d = from .50 to .79) and large (d = .80 and > .80) [28]. All participants who completed baseline measurements were included in the intention-to-treat analysis with the last observation carried forward used to replace missing data for each outcome [29]. Statistica v. 13.3 software and Excel spreadsheets were used for all statistical calculations.

## Results

The mean and SD of hurdle step kinematics for both groups during the acquisition and pos-testing phases are reported in Tables 1 and 2, respectively (see S1 Dataset).

**Table 1:** Mean and SD of kinematics achieved by both groups during acquisition phase.

Parameters	Group	Session 1	Session 2	Session 3	Session 4	Session 5
Hurdle running time	B	9.45±0.66	9.39±0.68	9.20±0.73	9.18±0.73	9.05±0.70^a^
	I	9.49±0.75	9.50±0.92	9.08±0.90^a^	8.90±0.69^a^	8.87±0.66^a^
Ground contact time*	B	0.196±0.016	0.194±0.015	0.190±0.017	0.192±0.015	0.192±0.010
	I	0.195±0.018	0.199±0.022	0.192±0.021	0.194±0.019	0.194±0.021
Hurdle flight time	B	0.458±0.045	0.460±0.039	0.455±0.044	0.449±0.043	0.445±0.038
	I	0.470±0.031	0.474±0.030	0.458±0.033	0.451±0.043^a^	0.451±0.043^a^
Hurdle step velocity	B	4.57±0.59	4.48±0.53	4.61±0.60	4.70±0.57	4.79±0.53^b^
	I	4.54±0.48	4.53±0.59	4.71±0.61	4.86±0.59^a^	4.87±0.59^a^
Hurdle step length	B	297.4±31.6	291.4±27.1	296.7±33.8	299.9±28.3	303.9±30.4^b^
	I	300.5±21.6	302.9±29.1	304.2±25.5	310.7±27.0	312.2±26.5^a^
Hurdle step angle	B	19.63±3.53	19.50±3.32	19.42±3.32	18.80±3.40	18.50±3.06
	I	19.87±2.97	19.98±3.60	18.98±3.49	18.57±2.99	18.06±3.61^a^

B-Blocked group, I-Increasing CI group

*:ground contact time before clearing the hurdle

^a^:significantly different from the first session (*p* < .05)

^b^:significantly different from the second session (*p* < .05).

**Table 2:** Mean and SD of kinematics achieved by both groups during retention and transfer tests.

Parameters	Group	Retention test after 10 days	Retention test after 40 days	Transfer test after 10 days	Transfer test after 40 days
Hurdle running time	B	9.01±0.62	9.07±0.64	9.03±0.62	9.07±0.73
	I	8.79±0.65	8.85±0.60	8.73±0.61	8.81±0.64
Ground contact time*	B	0.189±0.013	0.188±0.015	0.191±0.011	0.190±0.017
	I	0.190±0.022	0.190±0.017	0.190±0.020	0.190±0.019
Hurdle flight time	B	0.451±0.042	0.447±0.050	0.424±0.043	0.419±0.047
	I	0.448±0.043	0.446±0.036	0.418±0.040	0.426±0.039
Hurdle step velocity	B	4.81±0.47	4.82±0.52	4.88±0.46	4.88±0.53
	I	4.95±0.59	4.94±0.49	5.09±0.48	5.11±0.47
Hurdle step length	B	306.5±28.2	304.2±30.2	299.0±24.7	295.9±24.9
	I	312.7±23.2	313.3±23.2	307.3±15.8	312.8±14.0^a^
Hurdle step angle	B	18.10±2.97	18.20±3.56	16.76±2.96	16.54±3.30
	I	17.60±3.49	17.43±2.88	15.36±2.85	15.31±2.29^a^
Expert evaluation	B	1.82±0.52	1.93±0.58	1.85±0.55	1.90±0.59
	I	1.96±0.63	1.97±0.55	2.06±0.51	2.15±0.50

B-Blocked group, I-Increasing CI group

*:ground contact time before clearing the hurdle

^a^:significantly different from the Blocked group after 40 days (*p* < .05).

### Hurdle running time

There was a significant main effect for Time, *F*(4, 176) = 28.63, *p* < .001, η*_p_*^2^ = .39, and a main effect for Group x Time interaction, *F*(4, 176) = 3.29, *p* < .01, η*_p_*^2^ = .07 during the acquisition phase of the experiment. The post hoc analysis revealed that both groups significantly improved running time (*p* < .001), however this improvement occurred earlier in the Increasing CI group (in the third session, d = .49) and was greater (in the fifth session, d = .88) than in the Blocked group (in the fifth session, d = .59).

### Ground contact time before clearing the hurdle

Results of the ANOVA indicated a significant main effect for Time, *F*(4, 176) = 3.23, *p* < .05, η*_p_*^2^ = .07 during the acquisition phase, however no significant pairwise effects were evident when post-hoc tests were applied.

### Hurdle flight time

During acquisition, there was only a significant main effect for Time, *F*(4, 176) = 9.90, *p* < .001, η*_p_*^2^ = .18. Follow-up analysis showed that the Increasing CI group significantly (*p* < .05) decreased flight time during the fourth (d = .57) and fifth (d = .57) sessions relative to the first session.

### Hurdle step velocity

There was a significant main effect for Time, *F*(4, 176) = 14.32, *p* < .001, η*_p_*^2^ = .24 during acquisition. Post-hoc testing revealed that the Blocked group significantly (*p* < .05) increased step velocity during the fifth (d = .58) session compared to the second session, while the Increasing CI group significantly (*p* < .05) improved step velocity during the fourth (d = .59) and fifth (d = .61) sessions compared to the first session. The transfer test analysis showed a main effect for Group, *F*(1, 44) = 2.62, *p* = .11, η*_p_*^2^ = .06 indicating a trend toward statistical significance.

### Hurdle step length

There was a significant main effect for Time, *F*(4, 176) = 6.61, *p* < .001, η*_p_*^2^ = .13 during the acquisition phase of the study. Post-hoc testing found that the Blocked group significantly (*p* < .05) improved step length during the fifth session (d = .43) compared to the second session, while the Increasing CI group significantly (*p* < .05) increased step length during the fifth session (d = .49) compared to the first session. The transfer tests analysis yielded a significant main effect for Group, *F*(1, 44) = 4.83, *p* < .05, η*_p_*^2^ = .10, and a main effect for the Group x Time interaction, *F*(1, 44) = 5.18, *p* < .05, η*_p_*^2^ = .11. Post-hoc analysis found that the Increasing CI group had a significantly (*p* < .05, d = .84) longer step length than the Blocked group during the far transfer test 40 days following the end of practice.

### Hurdle step angle

There was a significant main effect for Time, *F*(4, 176) = 9.32, *p* < .001, η*_p_*^2^ = .17 during the acquisition phase. Post-hoc tests indicated that the Increasing CI group significantly (*p* < .05) decreased the step angle during the fifth session (d = .55) in comparison to the first session. In turn, the transfer tests analysis showed a significant main effect for Group, *F*(1, 44) = 6.30, *p* < .05, η*_p_*^2^ = .13. Follow-up analysis found that the Blocked group had a significantly (*p* < .05, d = .85) greater step angle than the Increasing CI group during the far transfer test.

### Expert evaluation

No significant main effects (*p* > .05) for Group, Time, or the Group x Time interaction were found during the retention or transfer tests, however the transfer tests analysis showed a main effect for Group, *F*(1, 44) = 3.77, *p* = .06, η*_p_*^2^ = .08 which approached statistical significance.

## Discussion

The present experiment aimed to investigate how practicing with systematic increases in CI facilitated the learning of sprint hurdles compared to practicing the same skill using a traditional blocked practice format within a PE context. Based on the current literature, we hypothesized that practicing with increasing CI would result in superior hurdling kinematics compared to practicing with a blocked arrangement of trials on the 10 day and 40 day retention and transfer tests.

The CI effect was not observed in the retention tests, but superior motor learning was facilitated within the Increasing CI practice group relative to the Blocked group during the 40 day delayed transfer test. Specifically, the results indicated that participants in the Increasing CI group more effectively cleared the hurdle, with a lower take-off step angle and longer hurdling step length compared to the Blocked group. In addition, the increasing CI practice schedule also produced higher hurdle step velocity and a better expert evaluation score during the delayed transfer test, but these differences did not meet the statistical criteria for significance and small and moderate effect sizes were identified, respectively. These results may indicate that the Increasing CI group adapted their hurdling technique more effectively over time, aligning with a key principle of motor learning that practice variability fosters an adaptable motor system [30]. The ability to adapt is critical for PE students who need to instruct a wide range of motor skills, often in novel conditions for effective teaching of PE in their future profession. The observed differences between the Increasing CI and Blocked groups in the delayed transfer test also underscore the potential long-term benefits of increasing CI practice in real-world contexts. Hence, while our experimental hypothesis was only partially supported, our findings underline the practical potential of implementing increasing CI schedules in PE teacher education programs.

Our findings demonstrate the benefits of utilizing an increasing CI practice schedule emerged only during the delayed transfer test. This finding is contrary to Barreiros [31] who reported the sensitivity to detect CI effects during transfer and retention tests are similar. We postulate a transfer tests could be more sensitive in detecting learning differences when the CI effect is examined within the learning of complex motor skills. This prediction was also proposed by authors studying the CI effect in volleyball skills [32]. Our research has shown that the CI effect can manifest several weeks following the conclusion of practice. We hypothesize the underlying mechanism of the CI effect is likely determined by a number of variables, including the complexity of the sport skill, learner experience level, as well as the length of time between the end of practice and retention and transfer testing, which is set arbitrarily in many motor learning studies. We believe that the use of post-tests at extended time intervals may exacerbate group differences and address some of the inconsistencies in the interpretation of the occurrence of the CI effect in motor learning research. The significant delay in the onset of the CI effect observed in this study highlights the need for research that looks more deeply into the phenomenon of long-term learning effects, including testing long-term memory [33].

A potential explanation for the lack of a CI effect during the retention tests was that the difficulty level of the sport skill used in this study was too high. Sprint hurdling is a much more complex skill with high nominal task difficulty [18] due to whole-body coordination and time constraints compared to skills used in previous CI research [15, 18]. A similar explanation was offered by Cheong et al. [23] who demonstrated that combinations of blocked and random practice were neither better nor worse than fixed low or high interference practice schedules when learning sport skills with a high level of difficulty. Interestingly, the authors of another study [34] involving children also indicated that a combination of blocked and randomized practice schedules did not have an unequivocally beneficial effect on hurdle running in children. However, Bortoli et al. [34] showed learning advantages when practicing a new complex sport skill (i.e., jumping). A factor which potentially affects learning outcomes in CI studies is the timing of the retention test. The amount of time between the cessation of practice and a retention test should be long enough to allow the impact of any performance variables to dissipate to best determine what was learned during practice (e.g. 10 and 40 days after acquisition was used in the present study). It is worth noting that a majority of CI research utilize a relatively short amount of time before a retention test, typically between 1–3 days following the cessation of acquisition [11, 13, 15]. Since the persistence of mastered skills among PE students is crucial to the nature of their future work, we believe that research aimed at optimizing sport skills in the curriculum of PE teachers should also take place with longer intervals between practice and post-tests to ensure that learned skills are retained beyond 1–3 days.

Another factor limiting the CI effect in motor learning studies [35, 36] may be related to the existence of a Generalized Motor Program (GMP) [36]. This concept proposes that a class of unique motor patterns sharing common invariant features (e.g., movement sequencing, relative timing structure, relative strength) which are stored in memory and are used every time the program is executed. Invariant features are aspects of a movement which are constant and do not change from one performance attempt to another. In contrast, the parameters (e.g., absolute force, time) of a movement may vary from one performance to another. In accordance with Magill and Hall [37], parameter modifications of the same GMP during acquisition may not provide sufficient interference in information processing to facilitate motor learning when measured on later retention and transfer testing. Although, the work reported by Porter and Magill [15] demonstrated that practicing with increases in CI resulted in enhanced learning effects for sport skills governed by the same GMP, the present results may support the view that practicing skills governed by the same GMP may reduce the occurrence of the CI effect [36]. These findings may also indicate that other amounts and types of task variations should be used in future studies to find more effective practice solutions for the learning of sprint hurdling. Consequently, additional motor tasks need to be tested to validate this conclusion and develop guidelines for applied practice.

The absence of a CI effect in the retention test can also be interpreted in the context of the learning assessment model used in this work. The most commonly used assessment design in motor learning research is to compare the learning outcomes of the experimental groups independently in the acquisition phase and retention test [35] rather than comparing the acquisition phase to the retention test. Hurdle running times were not different in the Blocked and Increased CI groups during the retention test. However, when baseline values of running time are considered, it was observed that the performance gains of the Increasing CI group were large (ES = 1.0, time difference between pre-test and retention test was 0.69 s), while the performance gains of the Blocked group were medium (ES = 0.69, time difference was 0.44 s). Therefore, it would be appropriate to consider using combined statistical analysis that include measurements from acquisition and post-testing to more fully evaluate learning effects. This calls for a more in-depth research methodology and data analysis in future motor learning studies.

Similar to previous research by Porter and Magill (Experiment 1) [15], no group differences were observed during acquisition, but both groups significantly improved sprint hurdle running time. This is contrary to the common CI effect where random practice depresses performance during acquisition relative to a blocked practice schedule. It is possible that preceding the increasing CI practice with blocked practice reduced the common CI effect during acquisition because the novice volunteers in our study achieved sufficient initial skill development during blocked practice allowing them to benefit from higher amounts of CI later in practice. It is noteworthy that during acquisition we observed desired changes in sprint hurdle kinematics (i.e., hurdle step velocity, flight time, step hurdle angle) only in the Increasing CI group. These results may suggest that a practice environment which challenges the learner with increasing levels of CI optimizes learning effects. These observations are in line with Gentile’s learning model [17] as well as Bjork’s [19, 20] concept of “desirable difficulties” Porter and Magill’s parallel development hypothesis [15], and the predictions of the “challenge point hypothesis” [18], all of which suggest that practice conditions should become progressively more demanding for learners as they are acquiring skill.

Although this study makes a unique contribution to the existing body of literature, this research is not free from limitations. The limitations of the present study offer avenues that need to be addressed in future investigations. First, the findings reported here may not be directly generalizable to athletes in a competitive setting. We tested low skilled athletes in the present study and all post-testing occurred outside the context of a track and field competition. It would be valuable from a practical and theoretical perspective to test the efficacy of practicing with an increasing CI practice schedule with more advanced athletes and measure their hurdling ability during track and field competitions under real-world constraints. Another potential limitation of this study is that it is possible that other organizations of sprint hurdling practice (e.g., using alternative hurdle heights, practicing alternative skills other than the high jump) may have resulted in findings different than those reported in the present experiment. It is clear that additional research within different applied settings and across diverse populations is needed to more fully understand how the CI effect influences the learning of complex motor skills [38].

In conclusion, having novices practice sprint hurdling following a progression of low to high CI in a PE setting seems promising, but still not conclusive. Specifically, when PE students mastered the fundamental level of sprint hurdling following a blocked practice schedule, which was later followed by a random arrangement of skills, a significant increase in variation of tasks was required to appropriately challenge learners as their skill was developed. The PE students utilizing an increase in CI may expect a maximized and more persistent learning effect relative to a traditional (blocked) practice schedule. These new findings have important implications for the planning and organization of instructional settings designed to facilitate the acquisition of sprint hurdles in university educational settings. Moreover, the results reported here have practical implications for PE teacher education programs by demonstrating that future teachers can learn sprint hurdling technique more effectively when practicing with increasing amounts of CI rather than static low CI. This is important because learning proper jumping technique is foundational for PE teachers which will have to repeatedly demonstrate the task to their future PE students. Providing a more technically sound demonstration will facilitate motor learning.

## Supporting information

S1 File Hurdle set up. (DOCX)

S2 File Examples of basic hurdle exercises. (DOCX)

S3 File Learning program. (DOCX)

S1 Dataset (XLSX)
