# Supplementary material for: The learning of sprint hurdles: A comparative study on increasing contextual interference and blocked practice schedules
Source: PLoS One. 2024 Jan 10;19(1):e0289916. doi: 10.1371/journal.pone.0289916 (PMC10781129; doi:10.1371/journal.pone.0289916)
Supplement: S1 File — (DOCX) [file pone.0289916.s001.docx]

**Supplement 1**

1. Detailed hurdle set up for the acquisition and retention test

The first hurdle was 13 m from the start line, the distance between consecutive hurdles was 8 m, and the distance from the last hurdle to the finish line was 5 m. The height of the hurdles was 0.91 m.

1. Detailed hurdle set up for the transfer test

The transfer test also involved 50 m sprint hurdles, but the first hurdle was 12.60 m from the start, the distance between the first and second hurdle was 8.40 m and the fifth hurdle was shifted towards the start line at a distance of 0.40 m. Thus, the distance between the fourth and fifth hurdles was 7.60 m. In addition, the height of the first and fourth hurdles was decreased to 0.84 m, and the height of the third hurdle was reduced to 0.76 m.
