# Supplementary material for: The learning of sprint hurdles: A comparative study on increasing contextual interference and blocked practice schedules
Source: PLoS One. 2024 Jan 10;19(1):e0289916. doi: 10.1371/journal.pone.0289916 (PMC10781129; doi:10.1371/journal.pone.0289916)
Supplement: S2 File — (DOCX) [file pone.0289916.s002.docx]

**Supplement 2**

Examples of basic hurdle exercises which were used during the first stage of the experiment:

1. Hip mobility and flexibility exercises

---Insert Figure 1a, 1b, 1c---

1. Wall exercises with the hurdle

---Insert Figure 2---

1. Walking over the hurdles

---Insert Figure 3---

1. Skipping beside the hurdles (drills for the lead and trial legs)

---Insert Figure 4a, 4b---

1. Sprints over lying sticks and mini-hurdles (height: 0.4-0.6 m) with a decreasing number of steps from five to three steps between hurdles

---Insert Figure 5---
