# Supplementary material for: The learning of sprint hurdles: A comparative study on increasing contextual interference and blocked practice schedules
Source: PLoS One. 2024 Jan 10;19(1):e0289916. doi: 10.1371/journal.pone.0289916 (PMC10781129; doi:10.1371/journal.pone.0289916)
Supplement: S3 File — (DOCX) [file pone.0289916.s003.docx]

**Supplement 3**

Table 1. Learning program for the Blocked and Increasing CI group

|  | **Blocked group** | **Increasing CI group** |
| --- | --- | --- |
|  | Repetition x Distance | Repetition x Distance |
| Session 1 | 9 x 13 m | 1 x 13 m , 1 x 21 m, 1 x 29 m, 1 x 21 m, 1 x 13 m, 1 x 21 m, 1 x 13 m |
| Session 2 | 7 x 21 m | 1 x 13 m, 1 x 29 m, 1 x 21 m, 1 x 13 m,  1 x 21 m, 1 x 13 m, 1 x 21 m |
| Session 3 | 6 x 29 m | 1 x 29 m, 1 x 37 m, 1 x 13 m, 1 x 29 m, 1 x 37 m, 1 x 29 m |
| Session 4 | 5 x 37 m | 1 x 37 m, 1 x 21 m, 1 x 29 m, 1 x 45 m,  1 x 13 m, 1 x 37 m |
| Session 5 | 4 x 45 m | 1 x 45 m, 1 x 13 m, 1 x 45 m, 1 x 37 m, 1 x 45 m |
